# The Approximate Number System Acuity Redefined: A Diffusion Model Approach

**DOI:** 10.3389/fpsyg.2015.01955

**Published:** 2015-12-24

**Authors:** Joonkoo Park, Jeffrey J. Starns

**Affiliations:** ^1^Department of Psychological and Brain Sciences, University of Massachusetts, AmherstMA, USA; ^2^Commonwealth Honors College, University of Massachusetts, AmherstMA, USA

**Keywords:** approximate number system, diffusion model, math ability, speed-accuracy tradeoff, Weber fraction

## Abstract

While all humans are capable of non-verbally representing numerical quantity using so-called the approximate number system (ANS), there exist considerable individual differences in its acuity. For example, in a non-symbolic number comparison task, some people find it easy to discriminate brief presentations of 14 dots from 16 dots while others do not. Quantifying individual ANS acuity from such a task has become an essential practice in the field, as individual differences in such a primitive number sense is thought to provide insights into individual differences in learned symbolic math abilities. However, the dominant method of characterizing ANS acuity—computing the Weber fraction (*w*)—only utilizes the accuracy data while ignoring response times (RT). Here, we offer a novel approach of quantifying ANS acuity by using the diffusion model, which accounts both accuracy and RT distributions. Specifically, the drift rate in the diffusion model, which indexes the quality of the stimulus information, is used to capture the precision of the internal quantity representation. Analysis of behavioral data shows that *w* is contaminated by speed-accuracy tradeoff, making it problematic as a measure of ANS acuity, while drift rate provides a measure more independent from speed-accuracy criterion settings. Furthermore, drift rate is a better predictor of symbolic math ability than *w*, suggesting a practical utility of the measure. These findings demonstrate critical limitations of the use of *w* and suggest clear advantages of using drift rate as a measure of primitive numerical competence.

## Introduction

The approximate number system (ANS) refers to a cognitive system that allows estimation of numerosities (i.e., cardinality of a set of items) without the use of language (Gallistel and Gelman, 1992; Dehaene, 2011). It is typically formalized as distributions of activation on a mental number line, where each numerosity is represented by a random variable with its mean and standard deviation as functions of that numerosity. Individual ANS acuity—the degree of precision of the internal quantity representation—is often conceptualized as the width of this distribution (see Piazza et al., 2004; Pica et al., 2004).

Recent findings demonstrating a relationship between ANS acuity and math ability have suggested a foundational role of this primitive cognitive system on later-learned math skills and thus have attracted scholarly and public interest in the ANS (e.g., Halberda et al., 2008; but for meta-analyses reporting negative findings, see Chen and Li, 2014; Fazio et al., 2014). Most often, individual ANS acuity is estimated from a numerosity comparison task. In this task, two arrays each containing a large number of dots are briefly presented, and participants are asked to quickly and accurately choose the array with more dots. Trials differ in the ratios between the two numerosities. In general smaller ratio trials (e.g., 11 vs. 10 dots) result in more errors while greater ratio trials (e.g., 16 vs. 8 dots) result in fewer errors. A typical practice for estimating individual ANS acuity assumes a specific psychophysical formulation (Piazza et al., 2004; Pica et al., 2004) and fits ratio-by-ratio accuracy data to the model in order to estimate the Weber fraction (*w*) as a measure of ANS acuity [but see Inglis and Gilmore (2014) for championing the use of raw accuracy].

This method of estimating ANS acuity, which dominates the field, has one critical limitation. Namely, the response time (RT) data are ignored. The few studies that have considered RT data used it either separately from accuracy or in combination with accuracy (e.g., inverse efficiency score; RT/accuracy) yet lacking theoretical connections to the ANS (for review and meta-analyses listing the dependent measures used in ANS studies, see Chen and Li, 2014; Fazio et al., 2014; Dietrich et al., 2015). Arguably, variability in accuracy and RT should be explained by the same mechanism in a perceptual decision-making task (for an argument in the context of numerical tasks, see Ratcliff et al., 2015). Therefore, analyzing accuracy in the absence of RT (or vice versa) may elicit a serious problem in assessing individual ANS acuity. For example, a participant might have a low *w* estimate because she focused on making fast decisions even if she had high ANS acuity.

Here, we introduce a novel approach of estimating individual ANS acuity by combining a sequential sampling model and an existing theoretical model of the ANS^1^. In particular, we use the well-established diffusion model (Ratcliff, 1978) to explain RT distributions of both correct and incorrect trials in a numerosity comparison task in terms of the rate of information accumulation (or “drift rate”), among many other model parameters. The drift rate is determined by the quality of the stimulus information, and thus it represents the quality of internal quantity representation in the present context. One advantage of the diffusion model is that it provides separate estimates of speed-accuracy tradeoff and evidence quality (e.g., Ratcliff and McKoon, 2008), while measures that are solely based on accuracy, such as *w*, are potentially influenced by participant-level variation in speed-accuracy settings. In this paper, we test whether individual differences in speed-accuracy tradeoff diminish the value of *w* as a measure of ANS acuity and then demonstrate the utility of using the drift rate as an estimate of ANS acuity.

## Materials and Methods

### Participants and Procedure

A total of 121 participants initially participated from the departmental research participation pool for course credit. Data from one participant, who made very few responses throughout the task, was immediately excluded. The resulting sample (*N* = 120) included 97 females with a mean age of 20.3 (range: 18.2–25.6) years. During a 1-h session, participants first performed an exact symbolic arithmetic task (see Section “Exact Symbolic Arithmetic”) followed by a numerosity comparison task (see Section “Numerosity Comparison”). The study procedure was approved by the University of Massachusetts Institutional Review Board. Participants gave written informed consent before participating in the study.

### Exact Symbolic Arithmetic

Participants solved two-operand addition and subtraction problems on a computer, similar to the tasks used in previous studies (Park and Brannon, 2013, 2014). During a 7-min block, participants were instructed to solve as many problems as possible using the number pad keys. The operands ranged from 11 to 195, and the correct answers ranged from 11 to 99. The arithmetic problems were randomly chosen from a larger set of problems, half of which contained either borrowing or carrying. The performance of this task (henceforth referred to as “math score” for simplicity) was quantified as the number of problems each participant solved correctly within the 7-min span. Data from five participants were not collected due to experimenter error.

### Numerosity Comparison

On each trial of this task, two dot arrays ranging in numerosity from 9 to 21 were presented for 750 ms on each side of a central fixation cross, after which only the fixation cross remained on the screen. Participants were asked to judge which side contained more dots as quickly and as accurately as possible by a manual (left or right index fingers) button press. The response was accepted from stimulus onset until 3 s after the stimulus onset, which was followed by an intertrial interval of 1.5 s before the onset of the following trial. The ratios between the two numerosities were 4:3, 7:6, 9:8, or 10:9. Dots within an array were homogeneous in size. In order to discourage reliance on a single other continuous variable when making numerical judgments, in half of the trials total surface area of the two dot arrays were equated, while individual surface area of each dot was equated in the other half. Independently of this manipulation, in half of the trials, density of the two dot arrays were equated, while the areas of an invisible circle that encompasses the dot array were equated in the other half. This means that the dot array parameters were systematically sampled from a parameter space represented by number (as one dimension) and two other dimensions orthogonal to number, namely size and spacing of the dot array. This design allows identical ranges of numerical and non-numerical cues (for more details, see DeWind et al., 2015; Park et al., 2015). Each block contained 64 trials, which took approximately 3.5 min. Each participant performed twelve blocks of a total of 768 trials with no trial-by-trial feedback.

### Weber Fraction Analysis

Models of the ANS assume that numerical quantity is represented by a distribution of activation on a mental number line (Van Oeffelen and Vos, 1982; Dehaene, 1992; Feigenson et al., 2004). ANS acuity is most often quantified by a Weber fraction (*w*) that quantifies the just noticeable difference as a fraction of stimulus value, which is conceptually the width of the activation distribution on the mental number line. Two specific forms have been proposed to represent the ANS: the linear model with scalar variability (e.g., Pica et al., 2004) and the logarithmic model with fixed variability (e.g., Piazza et al., 2004). While it has been argued that both models lead to similar predictions in participant’s behavioral responses (Pica et al., 2004), this is true only when accuracy is the only dependent variable. As explained below in Section “Alternative ‘Linear-Scale with Scalar Variability’ Model”, the linear model with scalar variability fails to match empirical patterns when both the accuracy and RT measures are considered together. Thus, in the current study, we use the logarithmic model with fixed variability to estimate individual numerical *w* (Piazza et al., 2004). In this model, the error rate in a comparison task is modeled as the area under the tail of a Gaussian curve with mean, log(*n*_L_)–log(*n*_S_), and standard deviation, √2*w*, as follows:

equation

where *n*_L_ is the larger and *n*_S_ is the smaller of the two numbers, and erfc(x) is the complementary error function. Split-half reliability coefficient after Spearman–Brown correction was computed from *w* estimated separately from even and odd runs.

### Drift Diffusion Model Analysis

#### Conceptual Overview of the Present Model

The diffusion model assumes that decisions are made by accumulating small samples of uncertain information until a threshold level of support for one response alternative is reached (Ratcliff, 1978). In the numerosity comparison task, the available responses are “left” and “right” for which side has the higher number of dots. Evidence accumulation begins at a starting point *z* between two response boundaries. The distance between response boundaries, *a*, represents response caution.

Evidence samples arrive from the stimulus and are variable across time, which here represents dynamic variability in the neural representation of quantity for each dot array. When the momentary quantity representation is higher for the left array, the accumulation process moves toward the “left” boundary, and vice versa. Accumulation continues until a boundary is reached, so wider boundaries produce slower responding (more evidence samples are needed) and more accurate responding (moment-to-moment variability in the accumulation process is less likely to make it hit the wrong boundary).

**FIGURE 1:**
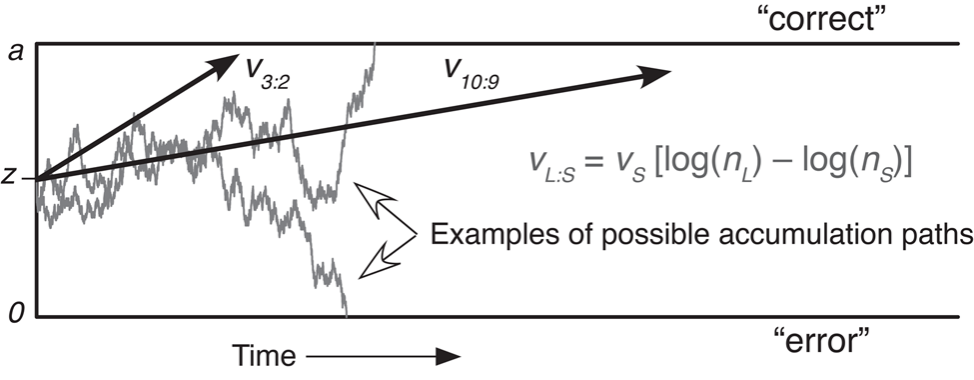
**A schematic illustration of the diffusion model in the context of a numerosity comparison task.** Here, trials with the larger number on the left and those with the larger number of the right are collapsed to represent boundaries for “correct” and “error” responses on each side. *n*_L_ is the larger and *n*_S_ is the smaller of the two numerosities. *v*_L:S_ represents the drift rate for each individual ratio condition.

The average rate of approach to a response boundary, or “drift rate,” represents the quality of evidence driving the decision. Here, drift rates were estimated simultaneously across four ratio conditions while assuming that numerosity is represented in a logarithmic scale. Specifically, the drift rate toward the correct boundary for each ratio condition was based on log(*n*_L_) – log(*n*_S_) multiplied by a scaling factor (henceforth referred to as drift scale), *v*_S_, that could vary across participants (see **Figure 1**). Thus, drift rates were higher for greater numerical ratios within a participant, and participants with higher values of *v*_S_ had higher drift rates across all ratio conditions, that is, better ANS acuity. The diffusion model assumes that drift rates follow a Gaussian distribution across trials within a condition, and the standard deviation of the distribution is measured by the η parameter (Ratcliff and McKoon, 2008). We held η constant across conditions, so our fits are consistent with a model in which numerosity representations have fixed variability (see Section “Alternative ‘Linear-Scale with Scalar Variability’ Model” for an alternative model). We estimated model parameters using the χ^2^ method described in Ratcliff and McKoon (2008). As in the *w* estimation, the split-half reliability coefficient of the drift scale was computed from the estimates from even and odd runs.

#### Model Fitting Details

The data were collapsed based on the ratio of dots on each side of the screen; for example, trials that had 12 dots on one side and nine on the other were combined with trials that had 16 dots on one side and 12 on the other. We also combined trials with the higher number on the left versus the right, with the response switched for the latter. Thus, the data for each participant included four conditions: ratios of 4:3, 7:6, 9:8, and 10:9. We found the 0.1, 0.3, 0.5, 0.7, and 0.9 quantiles of the RT distribution for both correct and error responses in each condition, and fit the model to the frequency of responses in the six RT bins defined by these boundaries. Thus, each condition contributed 12 response frequencies and 11 degrees of freedom (one response frequency is constrained to sum to the number of trials in the condition), resulting in 44 total degrees of freedom in the data across the ratio conditions. We estimated parameter values by minimizing a χ^2^ statistic computed with the empirical frequencies in each RT bin and the frequencies predicted by the model. When the total number of responses was between 2 and 8, we collapsed the data into two RT bins above and below the median RT. When the total number of responses was below 2, we collapsed the data into one bin representing the total frequency (i.e., RT data were not fit). The model had seven free parameters (also see Ratcliff and McKoon, 2008): the distance between the response boundaries (*a*), the range of the distribution of starting points across trials (*s*_Z_), the scaling factor on drift rate (*v*_S_, see **Figure 1**), the standard deviation of across-trial variation in drift rates (η), the mean duration of non-decision times (*T*_ER_), the range of the distribution of non-decision times across trials (*s*_T_), and the proportion of trials delayed by contaminant processes (*p*_O_). **Table 1** illustrates the summary statistics of these parameter estimates. These values are similar to previous applications of the model (e.g., Ratcliff and McKoon, 2008). The average starting point (*z*) was fixed at half of the boundary width for every participant.

**Table 1:** Mean and standard deviation of the diffusion model parameter estimates across participants.

	*a*	*s_Z_*	*v*_S_	η	*T*_ER_	*s*_T_	*p*_O_
Mean	0.093	0.023	1.163	0.167	0.414	0.219	0.004
Std	0.023	0.018	0.471	0.110	0.081	0.109	0.010


## Results

### Outlier Exclusion

In order to ensure that participants were attempting to perform the task and not just guessing on a large number of trials, participants with overall accuracy below 60% and/or more than 10% trials with reaction times less than 250 ms were excluded. These standards excluded 10 participants, leaving the final sample of *N* = 110. It should be noted that whether or not these 10 participants were excluded made no qualitative changes to the overall findings. In addition, the entire analysis on the *N* = 110 data set was performed again after excluding individual trials with RTs less than 250 ms (0.65% of the trials), but again the *w* and drift rate estimates were virtually identical. We thus report our analysis without individual trial exclusions.

### Weber Fraction Analysis

**FIGURE 2:**
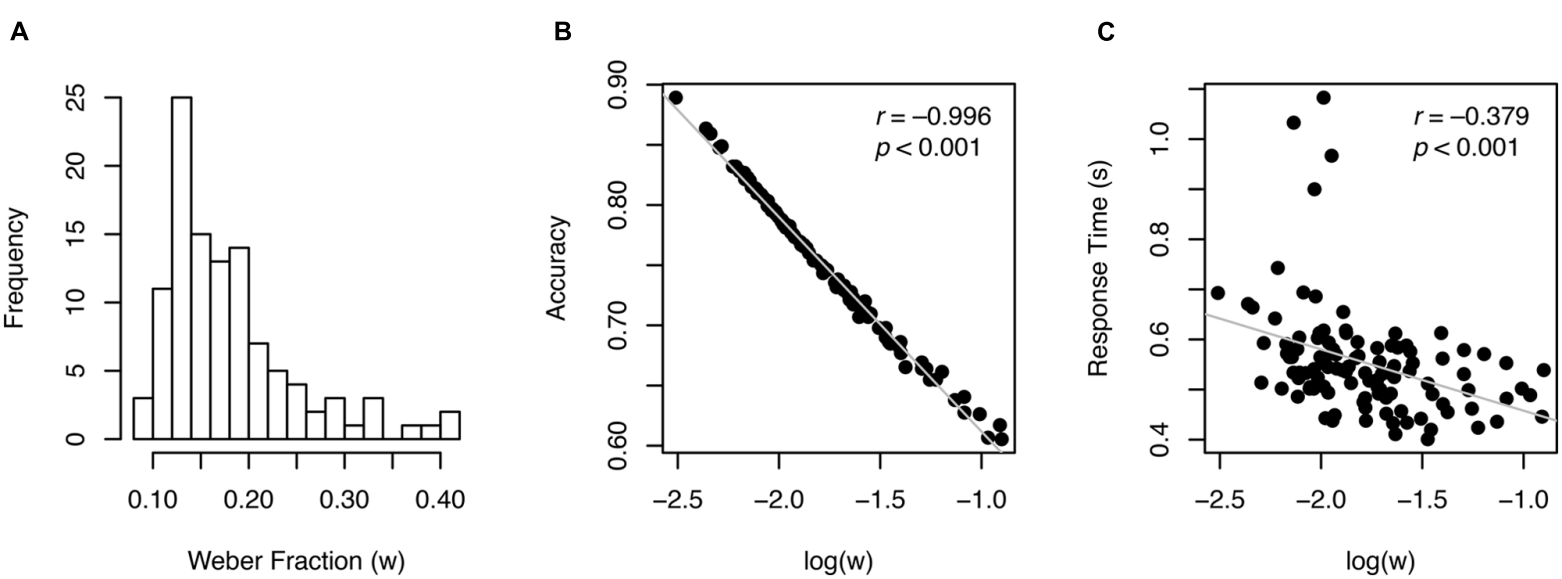
**Individual *w* measures (A) and their relationship with other variables (B,C).** Gray lines in scatterplots illustrate best linear fit.

Individual *w* values ranged from 0.081 to 0.41 with the median of 0.16, as shown in **Figure 2A**. Split-half reliability was 0.902, indicating that these estimates were highly reliable. Log-transformed *w* was almost perfectly correlated with accuracy (*r* = –0.996, *p* < 0.001, **Figure 2B**), indicating that *w* is a re-expression of accuracy, which is not surprising given how *w* is derived (Eq. 1; see similar patterns in previous work, e.g., Inglis and Gilmore, 2014).

Another behavioral pattern typically found in speeded perceptual decision-making task was revealed: accuracy and RT (reaction time) were significantly correlated (*r* = 0.391, *p* < 0.001) demonstrating speed-accuracy tradeoff across participants. As a corollary, RT was also highly correlated with log(*w*) (*r* = –0.379, *p* < 0.001, **Figure 2C**). These correlation patterns suggest that considering *w* exclusively to represent ANS acuity without taking RT into account may pose a serious problem, because better performance in terms of *w* could mean worse (slower) performance in terms of RT and vice versa.

### Drift Diffusion Model Analysis

**FIGURE 3:**
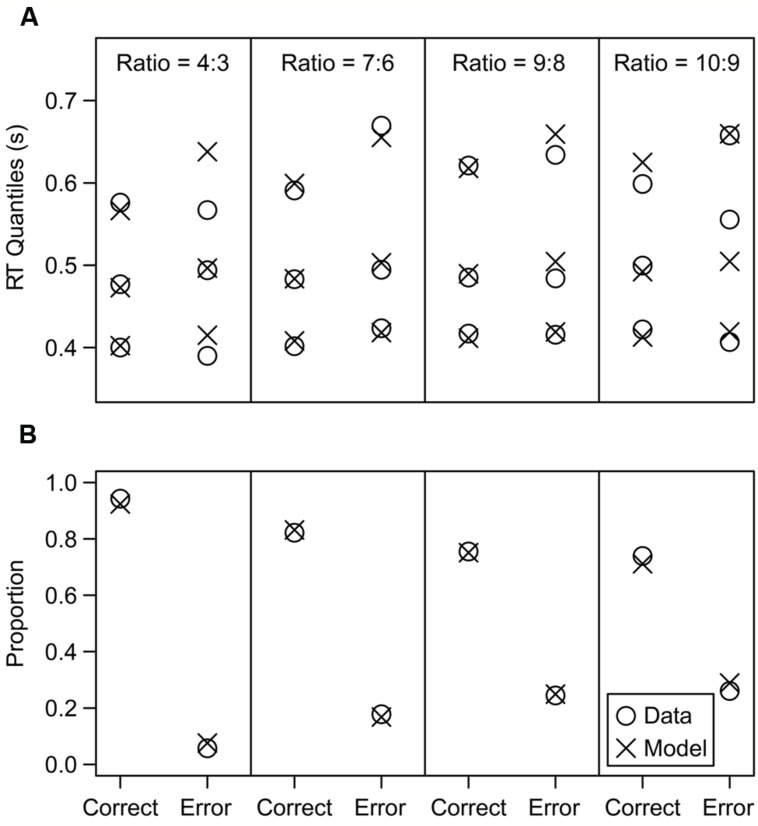
**Model fit results of an average participant (specifically, the participant whose fit criterion was closest to the median criterion across all participants).** **(A)** RT quantile fit results. The 0.1, 0.5, and 0.9 quantiles of each RT distribution are displayed (the model was also fit to the 0.3 and 0.7 quantiles, but they were omitted to reduce clutter). **(B)** Proportion fit results.

We first illustrate the typical model fit in **Figure 3**, which shows the fit for the participant who was closest to the median χ^2^ value across all participants. This participant had a χ^2^ value of 56.61, and the values across all subjects had a median of 56.58 and an inter-quartile range from 44.38 to 77.16. The model fit the proportion of correct and error responses very closely, and it also closely fit the RT distributions for correct responses, as shown by the 0.1, 0.5, and 0.9 quantiles of each distribution. The error RT distributions showed a poorer fit, but this is not surprising given that the distributions are estimated from a small number of error trials, especially in the higher accuracy conditions. Overall, the model successfully matched the data.

**FIGURE 4:**
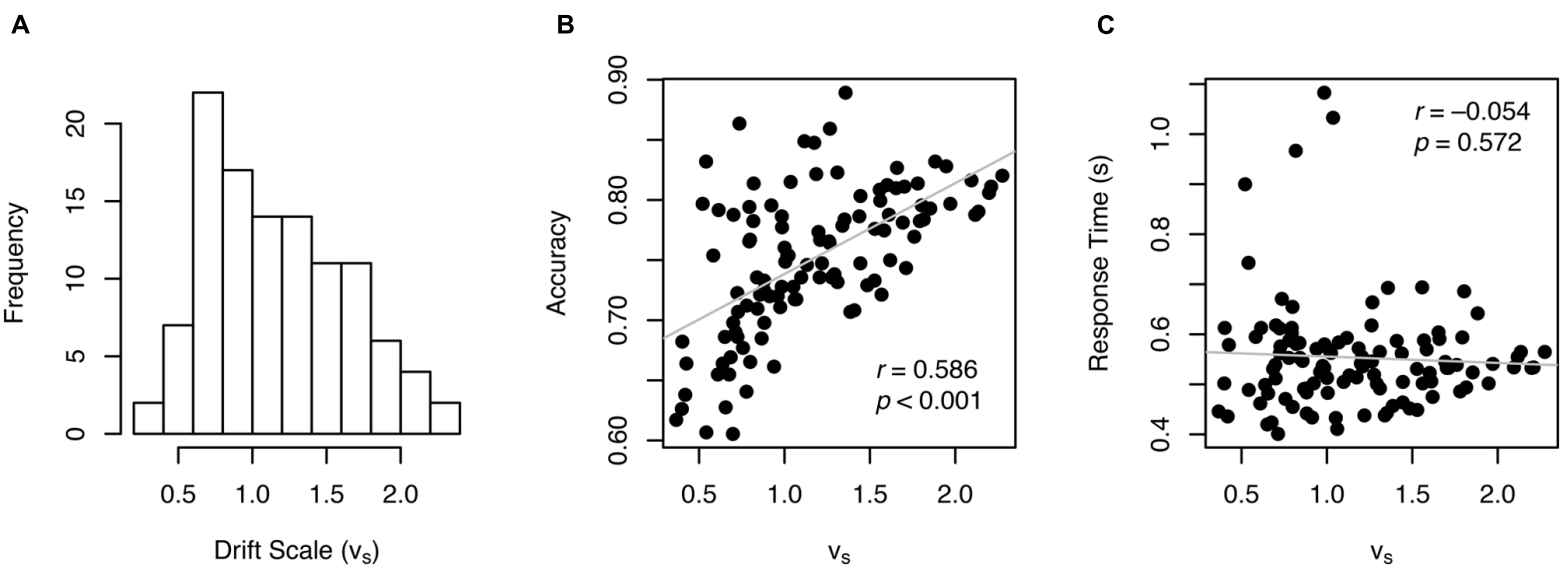
**Individual drift scale (*v*_S_) (A) and their relationship with other variables (B,C)**.

The drift scale parameter (*v*_S_) had a mean of 1.16 and ranged from 0.37 to 2.28 across individuals (**Figure 4A**) with a split-half reliability of 0.889. A drift scale of 1.16 produces drift rates of 0.33, 0.18, 0.14, and 0.12 for ratios of 4:3, 7:6, 9:8, and 10:9, respectively (see the formula in **Figure 1**). There was a high, but not a perfect, correlation between *v*_S_ and accuracy (*r* = 0.586, *p* < 0.001, **Figure 4B**), indicating that *v*_S_ is not a mere re-expression of accuracy. In addition, *v*_S_ was not correlated with RT (*r* = –0.054, *p* = 0.572, **Figure 4C**), which suggests that variability in other parameters (e.g., boundary width and non-decision time) primarily drove individual differences in RT.

**FIGURE 5:**
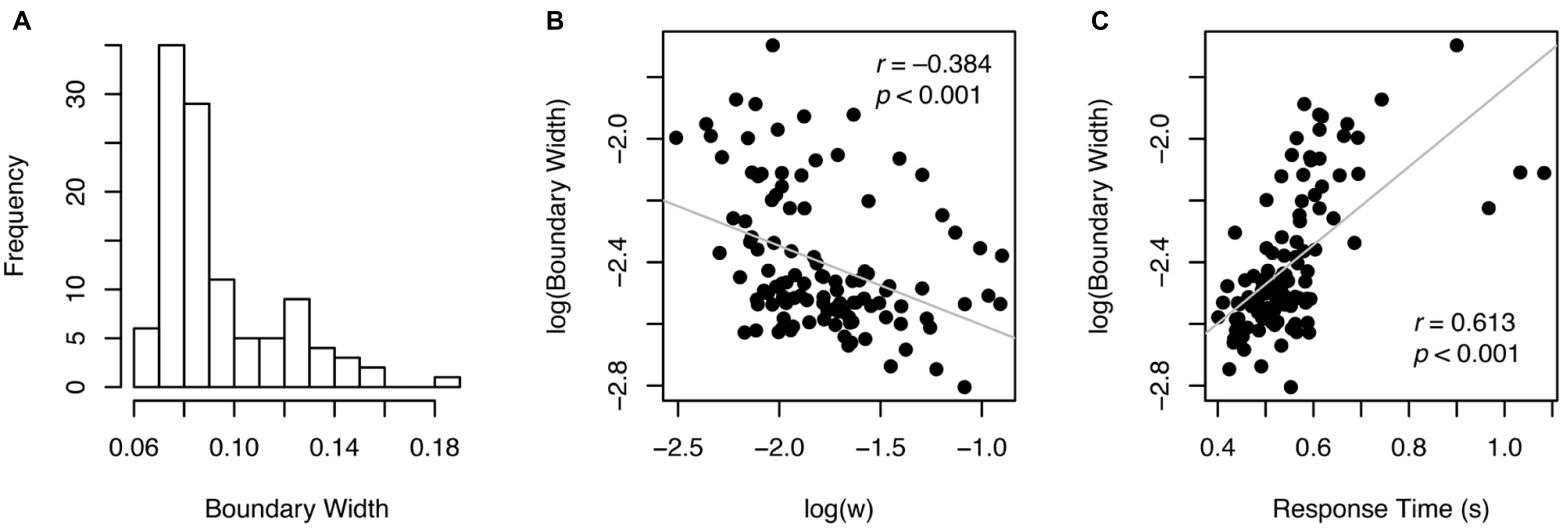
**Boundary width (A) and its relationship with *w* and RT (B,C)**.

Participants varied widely in terms of speed-accuracy tradeoff, with boundary width estimates ranging from 0.06 to 0.18 (**Figure 5A**). That is, some participants were making very quick decisions with little regard for accuracy (i.e., smaller boundary width), whereas others were being very cautious to avoid errors (i.e., greater boundary width). Thus, unsurprisingly, there was a strong correlation between *w* (accuracy) and log-transformed boundary width (*r* = –0.384, *p* < 0.001, **Figure 5B**). These results indicate that *w* is considerably influenced by individual adjustment of speed-accuracy tradeoffs. Boundary width (log-transformed) also correlated significantly with RT (*r* = –0.613, *p* < 0.001, **Figure 5C**).

### Alternative “Linear-Scale with Scalar Variability” Model

An alternative ANS model claims that the internal representation of numerosity varies linearly with the actual number of items, but the variance of this distribution increases for larger displays (linear-scale with scalar variability). These models make virtually identical predictions for accuracy (Pica et al., 2004); however, they can be distinguished with RT data. In our data, RTs increase as performance decreases across the ratio conditions, a pattern that was well fit by the log model (see **Figure 3**). In contrast, if the average drift rate remains constant and accuracy decreases are produced by increasing the across-trial variability in drift rates (η) as in the alternative model, RTs actually get *faster* as performance decreases (Starns and Ratcliff, 2014). Our data showed no evidence of such a speed up. For example, across our ratio and multiple conditions we can isolate two pairs of conditions for which the absolute difference between the number of items on each side remained the same but the total number of items increased substantially: 12:9 dots vs. 21:18 dots and 14:12 dots vs. 20:18 dots. For both pairs, accuracy was lower with a larger total number of items: 0.85 vs. 0.76 and 0.76 vs. 0.69, respectively. If this decrease was produced by increased variability with the same average drift rate, then RTs should also be faster as the total number of items increases. Instead, RTs (ms) were slower in both cases: 526 vs. 538 and 541 vs. 546. Therefore, only the log model, and not the variability-increase model, can simultaneously accommodate the accuracy and RT data.

### Correlation with Symbolic Arithmetic Abilities

**FIGURE 6:**
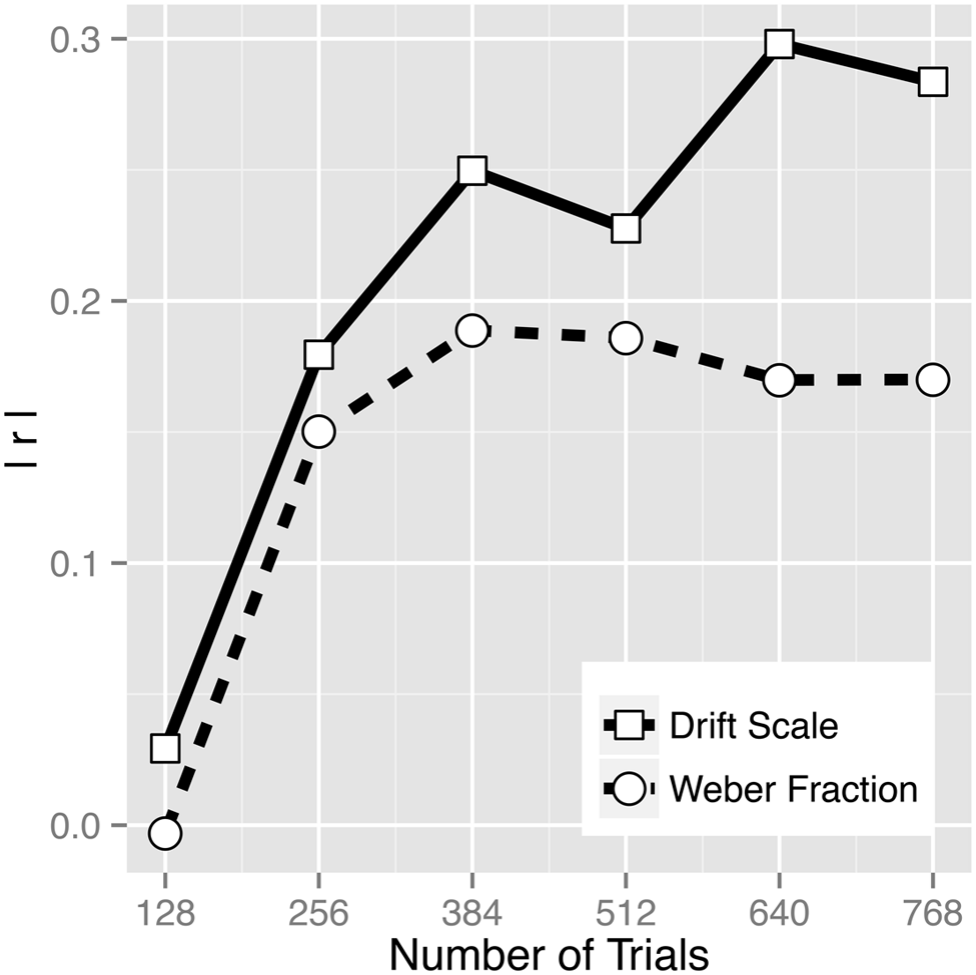
**Absolute value of the correlation coefficients between drift scale (*v*_s_) and math score and between Weber fraction (*w*) and math score as a function of number of trials**.

Correlation analyses revealed the association between math score and *v*_s_ (*r* = 0.284, *p* = 0.003) to be numerically greater than the association between math score and log(*w*) (*r* = –0.170, *p* = 0.083; **Figure 6**), demonstrating the superiority of the drift rate in predicting math abilities. Steiger’s (1980) test of the difference between two dependent correlations with one variable in common did not reach significance (*z* = –1.323, *p* = 0.189), but the superiority of the drift scale measure is also demonstrated by the strong correlation between boundary width and log(*w*) (**Figure 5B**). That is, whether a participant chooses to set conservative or liberal speed-accuracy criteria is independent of the quality of stimulus representation, and this factor adds noise to log(*w*) estimates (whereas the drift scale estimates come from a model that separately estimates evidence quality and speed-accuracy tradeoffs). Furthermore, in a multiple regression, *v*_s_ was found to be a significant predictor of math score even after controlling for log(*w*) that was entered as a covariate (*t*_102_ = 2.39, *p* = 0.019) and even after controlling for an inverse efficiency score (RT/accuracy; *t*_102_ = 2.98, *p* = 0.004). Note that inverse efficiency score itself did not correlate with math score (*r* = –0.042, *p* = 0.673), indicating that simply combining RT and accuracy does not yield a valid measure. In addition, the amount of variance in math ability explained by *v*_s_ was larger (*R*^2^ = 0.0804) than the amount of variance explained by log(*w*) and RT together (*R*^2^ = 0.0562), again indicating that the way diffusion model is capturing individual differences is more powerful than simply considering accuracy and RT data together. Drift scale having a better predictive power for math score was not an artifact of having unusually many trials in the task. As **Figure 6** illustrates, even when subsets of data were used to estimate the parameters, *v*_s_ consistently showed greater predictive power than *w*. Data from four to six 3.5-min blocks (256–384 trials) already showed a noticeable discrepancy in the predictive power. Note that greater predictive power of *v*_s_ than *w* consistently over the five data subsets (128, 256, 384, 512, and 640 trials) was not due to an artifact of *v*_s_ being a more reliable estimate, as the reliabilities across the data subsets were comparable between the two estimates (**Table 2**).

**Table 2:** Split-half reliability of the two estimates (Spearman–Brown corrected) across the data subsets.

**Number of trials**	**128**	**256**	**384**	**512**	**640**	**768**
*w*	0.48	0.66	0.75	0.87	0.88	0.90
*v*_s_	0.43	0.68	0.83	0.81	0.87	0.89


## Discussion

Estimating individual Weber fractions (*w*) has been the most widely used method to quantify the ANS acuity. This approach is firmly grounded in psychophysical theories and offers many advantages, including the fact that the measurement unit is independent from the actual stimulus parameters thus allowing direct comparisons between the results of different studies.

Nevertheless, this approach only utilizes accuracy information and ignores RT, which is a critical limitation from a standpoint that there is a considerable amount of speed-accuracy tradeoff during a perceptual decision-making task (see Ratcliff et al., 2015). In fact, *w* and RT were highly correlated in our dataset (see **Figure 2**). These results indicate that *w* may not represent only the fidelity of stimulus encoding and processing, as originally intended but it may also be influenced by individual adjustment of speed-accuracy tradeoffs.

One may argue that if there is small or no empirical correlation between accuracy and RT, it is valid to interpret that the two measures represent different mechanisms. For example, Halberda et al. (2012) claimed that *w* and RT scores were largely uncorrelated in their sample of over 10,000 participants and concluded that *w* and RT predict math scores independently^2^. Price et al. (2012) also found a significant correlation between a measure of *w* and RT in only one of the three numerosity comparison task variations. However, it should be noted that even if there is no apparent correlations between *w* (accuracy) and RT in a dataset, this does not necessarily mean that they are governed by independent mechanisms. For instance, in the diffusion model individual adjustment in speed-accuracy tradeoff is modeled as a single parameter (boundary width). This factor produces a positive correlation between accuracy and RT (more conservative speed-accuracy tradeoffs produce slower RTs and higher accuracy). In addition to this parameter, two other factors contribute to variability in RT and accuracy across participants: differences in the speed of evidence accumulation and differences in non-decision time. Speed of evidence accumulation produces a negative correlation between accuracy and RT (faster evidence accumulation produces faster RTs and higher accuracy), while non-decision time produces no relationship (non-decision time affects RT but not accuracy). Thus, even if speed-accuracy tradeoff is governed by a single mechanism (i.e., boundary width), a null correlation between accuracy and RT can easily be produced when all three factors are combined.

Taking this logic further, *v*_s_ may provide a more valid measure of ANS acuity than *w* by controlling speed-accuracy tradeoff even when the empirical correlation between accuracy and RT is small. We tested this idea by examining subsets of our own data where all factors (i.e., quality of stimulus representation, speed-accuracy tradeoff setting, non-decision time) balance out to give no or small overall correlation between accuracy and RT. Specifically, in each of 100,000 repetitions, we randomly selected half of the subjects (*N* = 55) and recorded the correlation between accuracy and RT. Nine hundred and forty-four of the 100,000 cases resulted in a non-significant correlation (*p* > 0.05). In these 944 cases, we then computed the correlation between *w* and math as well as between *v*_s_ and math. In 877 of the 944 cases (93%), the correlation between *v*_s_ and math was greater than the correlation between *w* and math, providing empirical support to the idea that modeling out the effects of speed-accuracy tradeoff may be beneficial in estimating ANS acuity even in the absence of correlation between accuracy and RT.

It should be noted that there have been recent advancements in improving the traditional way of estimating the ANS acuity (i.e., *w*; e.g., DeWind et al., 2015; Odic et al., 2015); however, these methods do not account for RT. In addition, previous studies using a numerosity comparison task used mean RT, an RT difference under different numerical ratios, or a combination of RT and accuracy in order to quantify individual performance (to list a few, Holloway and Ansari, 2009; de Oliveira Ferreira et al., 2012; Sasanguie et al., 2012); however, these approaches lack a clear connection to the theoretical model of the ANS, making it difficult to conceptualize what the dependent measures really mean.

In this study, we used a theory-based sequential sampling model in combination with the theoretical model of the ANS to offer a new approach of estimating ANS acuity. To be specific, a novel variant of the diffusion model (Ratcliff, 1978) was developed to assess the quality of the internal quantity representation by incorporating both the accuracy and RT distributions. This approach allows researchers to factor out the effects of speed-accuracy tradeoff settings (boundary width) from the estimation of the quality of stimulus information (drift scale, *v*_S_). Indeed, the results from our data showed a strong association between boundary width and both accuracy and RT, suggesting using *w* (accuracy alone) as the ANS acuity may not be ideal given that *w* is influenced by an individual tendency to stress accuracy over speed. Moreover, there was very little, if any, correlation between *v*_S_ and RT (see **Figure 4**), suggesting that the measure of internal quantity representation using the diffusion model is much less influenced by speed-accuracy tradeoffs than *w*.

In addition to factoring out speed-accuracy tradeoff settings, one other potential advantage of diffusion modeling is that it reliably factors out non-decision time (i.e., encoding and response output time) from the quality of internal quantity representation. Therefore, it could more effectively estimate ANS acuity in various subject groups (e.g., children or older adults) who potentially show a great amount of variability in non-decision time. These investigations should be an important agenda for future research.

On a theoretical note, our results suggest that RT modeling can inform theoretical accounts of number estimation. If one considers only accuracy data, then two underlying models make identical predictions for the change in performance across ratios: either number representations have a logarithmic relationship to actual numerosities with a constant variance or a linear relationship with increasing variance. However, these accounts make opposite predictions for RT when they are translated into diffusion model drift rates: the first model predicts slower RTs for conditions with lower accuracy and the second predicts faster RTs. We found that the logarithmic model closely fit both accuracy data and RT distributions. In contrast, our data showed no evidence of faster RTs for conditions with a higher total number of items. We cannot strongly rule out the possibility that drift variability changes across conditions, but this factor alone cannot explain performance changes across the ratio conditions. The important point is that the logarithmic model provides a good fit to data, and this model has the added benefit of offering an easily interpretable measure of overall ANS acuity (drift scale).

Approximate number system acuity is most often used to investigate how individual differences in numerical quantity representation explain other variables using correlational analyses (e.g., see Section “Correlation with Symbolic Arithmetic Abilities”). Thus, it is important to understand the relative reliabilities of *w* and *v*_S_ estimates. On the one hand, because diffusion model includes many more parameters than the Weber fraction estimation, one may reason that *v*_S_ may be less reliable than *w*. On the other hand, because diffusion model additionally includes RT data, one may reason that *v*_S_ may be more reliable than *w*. As reported, split-half reliabilities of *w* and *v*_S_ were comparable between the two at least in our dataset from young adult participants, suggesting that the drawback of having more parameters to fit in the diffusion model is overcome by including more data in the model. Note that comparable reliability scores between the two estimates suggest that, under the situation where a researcher attempts to correlate ANS acuity with another variable, using *v*_s_ instead of *w* might be a wiser choice given that there is a potential advantage in validity without a sacrifice in reliability. Based on our sample, regardless of whether *w* or *v*_s_ will be estimated, collecting 256 total trials or more is recommended for reasonably reliable estimates, although it should be noted that a reliability near 0.50 under 128 trials is not the worst case scenario in typical cognitive measures (e.g., see Maloney et al., 2010). Researchers interested in applying the diffusion model to estimate ANS acuity as in our study are encouraged to take advantage of diffusion model toolboxes that are readily available (Vandekerckhove and Tuerlinckx, 2007; Wagenmakers et al., 2008; Voss et al., 2015).

We have so far described the advantage of the diffusion model-based approach to estimating ANS acuity from a perspective of a perceptual decision-making process (i.e., factoring out speed-accuracy tradeoff). One important remaining question is which of the two measures (*w* or *v*_s_) is a more valid measure of ANS acuity? This is not an easy question to ask, not just for *w* or *v*_s_ but for any estimate that claims to measure ANS acuity because ANS is a hypothetical cognitive construct and there is no obvious way to test its validity.

Here, we took the approach to exploit one of the most influential, yet hotly debated, propositions in the ANS literature. That is, ANS is proposed to be an important foundation for symbolic mathematical abilities (Gallistel and Gelman, 1992; Halberda et al., 2008; Dehaene, 2011). Supporting this idea, studies have reported a correlation between ANS acuity (mostly estimated in *w* or accuracy) and math scores (for meta-analyses that also include negative findings, see Chen and Li, 2014; Fazio et al., 2014). Thus, according to this proposition, if one of the two measures discussed so far (*w* or *v*_s_) is a better estimate of ANS acuity, exact symbolic arithmetic performance should be better predicted by that measure than by the other. Our results show that *v*_s_ is a better predictor for symbolic arithmetic performance, lending support to the idea that is a *v*_s_ more valid measure than *w*.

One limitation of the present approach (as well as the traditional approach of estimating *w*) is that it does not account for the effects of non-numerical cues in the stimuli. Because the number of items in an array is necessarily confounded with other cues such as the size of each item or the density of the array, it is impossible to assess the effect of numerical processing independent of all other non-numerical cues. Many studies now show that participants’ performance in a numerosity comparison task is influenced by such non-numerical cues (for recent reviews, see Leibovich and Henik, 2013; Dietrich et al., 2015). Thus, one needs to be cautious in interpreting both *v*_s_ and *w* as an index of numerical acuity. As we have shown, *w* measures have an additional layer of process contamination in that they are influenced by speed-accuracy tradeoffs.

However, there are a few reasons to believe that participant’s judgment in the present numerosity comparison task is based more on numerical than other non-numerical cues. First, DeWind et al. (2015) have recently developed an innovative technique to statistically isolate the unique effects of numerical and non-numerical cues during a numerosity comparison task. Using stimulus parameters that are systematically constructed to span comparable ranges of numerical and non-numerical cues (as in our design), they found that numerosity was the primary dimension driving the participants’ behavioral decision-making process. Second, in a series of EEG experiments, participants passively viewed dot arrays that were systematically constructed to assess the role of numerical and non-numerical cues using the same technique (Park et al., 2015). Even though there was no emphasis on numerosity, participants’ visual evoked potentials were most sensitive to the modulation of the numerosity dimension than to any other dimensions. These results suggest that numerosity is the primary dimension that is directly encoded in the visual system. Thus, it is plausible that the quality of the stimulus representation in the present diffusion model is based on the numerical quantity. Nevertheless, an important next step would be to extend our current diffusion model to incorporate the effects of non-numerical cues (as in DeWind et al., 2015 and Park et al., 2015).

In summary, the ANS is thought to serve as an important foundation for symbolic mathematical ability (Gallistel and Gelman, 1992; Dehaene, 2011). Assessing ANS acuity has been a critical first step for testing the link between primitive number sense and math performance and for generating further hypotheses about behavioral and educational implications of ANS. While computing a numerical Weber fraction (*w*) has been a dominant way of estimating the ANS acuity in the field, the results from our data from young adult participants demonstrate that individual *w* estimates are largely influenced by speed-accuracy tradeoffs (**Figures 2** and **5**), questioning the validity of the measure. Our approach of using a drift diffusion model illustrates that drift rate captures the quality of the non-symbolic numerical quantity information presumably with less influence from task-specific variables that affect individual speed-accuracy tradeoffs (also see Ratcliff et al., 2015). Our novel drift scale measure thus may be a better measure of primitive numerical competence than the widely used *w*, also indicated by a greater predictive power for symbolic arithmetic ability (**Figure 6**).

## Author Contributions

JP devised the study and performed the experiment. Both authors analyzed data, interpreted results, and wrote the manuscript.

## Conflict of Interest Statement

The authors declare that the research was conducted in the absence of any commercial or financial relationships that could be construed as a potential conflict of interest.

## Footnotes

1 While preparing this manuscript, we learned via a conference presentation that Ratcliff and McKoon (2015) are pursuing a very similar approach.

2 But note the correlation between *w* and math scores (*r* = –0.19) and between RT and math scores (*r* = –0.09) were not much stronger or even weaker than the correlation between *w* and RT (*r* = –0.11).
